# Chemical Composition and Antifungal and Antibiofilm Effects of *Vitex pseudo-negundo* Essential Oil against Pathogenic Fungal Strains

**DOI:** 10.1155/2023/3423440

**Published:** 2023-10-03

**Authors:** Zahra Zareshahrabadi, Mohammad Jamal Saharkhiz, Maryam Izadpanah, Aida Iraji, Maryam Emaminia, Maryam Motealeh, Hossein Khodadadi, Kamiar Zomorodian

**Affiliations:** ^1^Basic Sciences in Infectious Diseases Research Center, Shiraz University of Medical Sciences, Shiraz, Iran; ^2^Medicinal Plants Processing Research Center, Shiraz University of Medical Sciences, Shiraz, Iran; ^3^Department of Horticultural Sciences, Faculty of Agriculture, Shiraz University, Shiraz, Iran; ^4^Department of Parasitology and Mycology, Shiraz University of Medical Sciences, Shiraz, Iran; ^5^Research Center for Traditional Medicine and History of Medicine, Department of Persian Medicine, School of Medicine, Shiraz University of Medical Sciences, Shiraz, Iran; ^6^Central Research Laboratory, Shiraz University of Medical Sciences, Shiraz, Iran; ^7^Shiraz University of Medical Sciences, Shiraz, Iran; ^8^Cellular and Molecular Biology-Genetics, Shiraz University of Medical Sciences, Shiraz, Iran

## Abstract

**Background:**

*Vitex pseudo-negundo* is a plant of the *Lamiaceae* family that grows in different parts of the world and the vicinity of seasonal rivers in Iran.

**Methods:**

The chemical composition of the *Vitex pseudo-negundo* essential oils was distilled and evaluated using gas chromatography/mass spectrometry. The antifungal activity of the essential oils against the fungal strains was analyzed by broth microdilution methods as suggested by the Clinical and Laboratory Standards Institute. Furthermore, the antibiofilm activity of the *Vitex pseudo-negundo* essential oils was assessed using the XTT reduction assay.

**Results:**

Based on GC/MS analysis, the major components of the *Vitex pseudo-negundo* essential oils were *α*-pinene, *α*-terpinyl acetate, limonene, and (E)-caryophyllene. The growth of tested yeasts was inhibited at concentrations ranging from 2 to 64 *μ*l/mL. *Vitex pseudo-negundo* fruit essential oil was the most effective in inhibiting yeast growth. Moreover, the essential oils exhibited antifungal activity against filamentous fungi strains. Additionally, the biofilm formation of *Candida albicans* was inhibited by the leaf, flower, and fruit of the essential oils.

**Conclusion:**

Considering the significant antifungal activities of these essential oils, they can be considered a potential source for formulating novel agents to control fungal infections.

## 1. Introduction

*Vitex pseudo-negundo* belongs to the *Lamiaceae* family and is commonly known as the Chinese chaste tree or five-leaved chaste tree. *Vitex pseudo-negundo* is typically characterized by a large aromatic square shrub and thickly whitish, containing a multitude of phytochemical compounds [1]. There are approximately 270 species of this genus which are distributed in regions around the world from the Mediterranean areas to Central Asia [2]. This genus serves as an important natural source of traditional medicine in Western Asia, including Syria, Iran, Afghanistan, and other areas. In Iran, it can be found in Tehran, Karaj, Qom, Khorasan, Ahvaz, and Fars provinces [1, 3, 4]. Recent studies have revealed that *Vitex pseudo-negundo* possesses various medicinal properties such as antimicrobial, anti-inflammatory, and antioxidant activities. It has been used to treat several female disorders including endometriosis, abnormal menstrual cycles, menopausal dysfunction, and inadequate lactation. On the other hand, the incidence of fungal infections is currently on the rise, placing significant pressure on healthcare professionals [5, 6].

One of the most important reasons for this increase is related to the rising number of patients with immune system defects resulting from changes in medical practice, including chemotherapy and immunosuppressive drugs. Additionally, invasive fungal infections (IFI) have significantly increased among immunosuppressant patients, especially among those who are HIV-infected [7]. The *Candida* genus comprises many species considered normal flora of mucosal surfaces. This species has been regarded as the most common pathogenic yeast and also the fourth cause of systemic infections in hospitalized patients. Furthermore, it is worth noting that recent clinical implications have highlighted the increasing involvement of non-*Candida albicans* species [8].

Among the filamentous fungi, *Aspergillus* species are known as common saprophytes found in various ecological niches. Among the 200 identified species in this genus, *A. fumigatus* and *A. flavus* are considered important human pathogenic species, causing various forms of the disease [9]. Another group of significant filamentous fungi is dermatophytes, which are keratinophilic fungi encompassing three major types: *Trichophyton*, *Epidermophyton,* and *Microsporum* [10]. Due to the widespread use of broad‐spectrum antifungal drugs, there has been a noticeable increase in drug resistance [8, 11–13]. Consequently, there is a high demand for the development of new and effective antifungal agents with low toxicity [14, 15]. Nowadays, there is a growing preference to alternative-based natural compounds with lower resistance rates [16].

Essential oils (EOs) as secondary metabolites obtained from the aerial part of plants exhibit various beneficial biological effects, including antimicrobial, anti-inflammatory, antitumor, and analgesic activities [17–19]. To our knowledge, there are a limited number of studies on the antibacterial activities of *Vitex pseudo-negundo*; however, there have been no reports on the antifungal and antibiofilm activities of these mentioned EOs. Therefore, the present study was designed to characterize the chemical composition of the EO distilled from the leaf, flower, and fruit of *Vitex pseudo*-*negundo*, collected from the Fars province in Iran, and evaluate its antifungal and antibiofilm activities.

## 2. Materials and Methods

### 2.1. Plant Material and EOs Isolation

*Vitex pseudo-negundo* was collected at the flowering stage from the forest area in Fasa city, Fars province, Iran. The leaves, flowers, and fruits of the plant were authenticated, and a voucher specimen (HSU 3005) was deposited in the Herbarium of Shiraz University, Shiraz, Iran. Fifty grams of the air-dried parts of *Vitex pseudo-negundo* was hydrodistilled for 3 hours using a Clevenger-type apparatus. The obtained essential oils (EOs) were then dried over anhydrous sodium sulfate and stored in brown vials at 4°C until further investigations [20].

### 2.2. Gas Chromatography/Mass Spectrometry (GC/MS)

The GC/MS analysis was performed using an Agilent 7000 triple quad mass spectrometer (Agilent Technologies, USA) coupled with an Agilent 7890A gas chromatography (Agilent Technologies, USA) instrument. The oven temperature was initially set at 70°C and then ramped up to 280°C, where it was held for 4 minutes. The injector and auxiliary temperatures were maintained at 250°C and 280°C, respectively. The division ratio was 1/50, and the carrier gas, helium, flowed at a rate of 1.2 ml/min. The operating parameters of the mass spectrometer were as follows: ionization voltage at 70 eV and a mass range of 40–650 atomic mass units (amu) [16]. Retention indices (RIs) were determined for all compounds using the retention times of n-alkanes (C6–C24) injected after the oil under the same conditions. The compounds were identified by comparing their RIs with those reported in previous studies (Adams, 2007) and by matching their mass spectra with the spectra in the Wiley library.

### 2.3. Determination of Antifungal Activities

#### 2.3.1. Microorganisms and Culture Conditions

Ten American Type Culture Collection (ATCC) and Centraal Bureau voor Schimmelcultures (CBS) strains of fungi including *Candida albicans* (ATCC 10261), *C. tropicalis* (ATCC 750), *C. krusei* (ATCC 6258), *C. glabrata* (ATCC 90030), *C. dubliniensis* (CBS 8501), *C. parapsilosis* (ATCC 4344), *Cryptococcus neoformans* (ATCC 9011) *Aspergillus flavus* (ATCC 64025), *A. clavatus* (CBS 514.65), and *A. fumigatus* (ATCC14110) were used for the evaluation of the EOs antifungal activities. Furthermore, the antifungal activities of the EOs were examined against clinical isolates of *Microsporum gypseum, Epidermophyton floccosum,* and *Trichophyton rubrum* also, 6 clinical isolates of *C. albicans* that were susceptible (3 isolates) and resistant (3 isolates) to fluconazole were studied. Also, fluconazole as a commercial antimicrobial was chosen as a control.

The mentioned yeasts and molds fungi were subcultured on Sabouraud dextrose agar (SDA, Merck, Germany) and potato dextrose agar (PDA, Merck, Germany), respectively. The culture plates were incubated to inhibit the bacteria growth, and 50 mg/L chloramphenicol (Sigma-Aldrich, Germany) was added to the culture media of fungi [11].

#### 2.3.2. Antifungal Susceptibility Tests

The determination of minimum inhibitory concentrations (MICs) of the EOs against the tested fungi was performed using the broth microdilution method, following the guidelines of the Clinical and Laboratory Standards Institute (CLSI). Serial dilutions of the EOs (ranging from 0.012 to 64 *μ*l/mL) were prepared in 96-well microtiter plates using RPMI-1640 media (Sigma, St. Louis, USA). The inoculum yeast suspensions were adjusted to a turbidity equivalent to a 0.5 McFarland standard (approximately 1–5 × 10^6^ cells/mL) using a spectrophotometer at a wavelength (*λ*) of 600 nm. For molds, conidia were collected, and their suspensions were adjusted to a turbidity equivalent to a 0.5 McFarland standard (approximately 1–5 × 10^6^ conidia/mL) using a spectrophotometer at a specific wavelength (*λ*) of 530 nm. Working suspensions of the studied yeast and mold fungi were prepared in 1/1000 and 1/50 dilutions with RPMI, respectively. After adding 0.1 mL of the prepared inoculum to each well, the plates were incubated at 32°C for 24–48 hours and at 25°C for 48–72 hours for yeast and molds, respectively. The wells of the first column were considered as negative control, and there were also growth controls containing medium without EO. The MIC was defined as the lowest concentration of EOs that showed visible growth reduction. Additionally, for the determination of the minimum fungicidal concentration (MFC), 10 *µ*L of the media from the wells showing no visible growth was cultured on SDA for fungi. The MFCs were defined as the lowest concentration of EOs showing no growth or fewer than 4 colonies. Each experiment was conducted in triplicate [20–23].

### 2.4. Determination of the Biofilm Inhibitory Activity

#### 2.4.1. Biofilm Preparation and Growth

To form biofilms, standard strains of *C. albicans* (CBS 5982) were cultured on SDA plates. After 48 hours, one loop of *C. albicans* colonies was transferred to Sabouraud dextrose broth (SDB, Merck, Germany) and incubated for 24 hours at 32°C on a shaker set at 100 rpm. Yeast cells were then harvested twice with sterile phosphate-buffered saline (PBS) and resuspended in RPMI 1640. The cell concentration was measured spectrophotometrically, resulting in a concentration of 1 × 10^6^ cells/mL at a wavelength (*λ*) of 600 nm. Serial dilutions of EOs (ranging from 0.012 to 64 *μ*L/mL) were prepared in 96-well microtiter plates using RPMI 1640 medium. After adding 0.1 mL of yeast, the plates were incubated at 30°C for 48 hours. Additionally, 200 *μ*L of culture medium alone was used as a negative control, and RPMI with yeast without EOs served as a positive control [24].

#### 2.4.2. Assessment of Biofilm Formation

Biofilm formation was assessed using the 2,3-bis(2-methoxy-4-nitro-5-sulfophenyl)-2H-tetrazolium-5-carboxanilide (XTT) reduction method. After 48 hours of incubation, the biofilms were washed twice with sterile PBS to remove nonadherent cells. Subsequently, 100 *μ*L of XTT menadione solution (Sigma Chemical Co., St. Louis, USA) was added to each well. The 96-well microtiter plates were then incubated at 32°C for 4 hours in a dark environment. Finally, colorimetric changes were measured using a microplate reader.

### 2.5. Statistical Analysis

Results were reported as means ± standard deviation. All assays were performed considering three analytical replications and the graphs were plotted using GraphPad Prism 6 software (GraphPad Software, Inc., San Diego, CA, USA).

## 3. Results and Discussion

### 3.1. EOs Content

EOs chemical compositions of aerial parts of *Vitex pseudo-negundo* (leaf, flower, and fruit) are shown in Table 1. In the present study, 50, 55, and 56 compounds were identified in leaf, flower, and fruit, comprising 98.84%, 98.82%, and 97.74% of the total composition of the EOs, respectively.

The major identified constituents of the leaf presented in this study were *α*-pinene (39.3%) followed by *α*-terpinyl acetate (17.5%), limonene (13.8), and (E)-caryophyllene (7.2%) which represented approximately 78% (by GC peak area) of the total composition. The leaf oil composition presented in this study differed from the previous study reporting 1,8-cineole (30∼50%) [25, 26] and 2,6,6-trimethylbicyclo [3.1.1] hept-2-ene (18.5%) [27] as the main compounds of mentioned EO. Results on the EO composition of the leaf are in agreement with Salehpour et al. [1] and Mohammadi et al. [20] demonstrating *α*-pinene as a major compound in the fresh leaf of *Vitex pseudo-negundo*.

Besides, the major compounds of *Vitex pseudo-negundo* flower EO were *α*-pinene (30.3%), followed by *α*-terpinyl acetate (17.3%), (E)-caryophyllene (13.1%), and limonene (11.6) which comprised more than two-thirds of the EO of the plant flower. The results of the present investigation partially match with the results of earlier findings reported that *α*-pinene (25.4–44.2%), *α*-terpinyl acetate (1.1–17.3%), and limonene (6.9–11.9) are the major monoterpenoids obtained from the flowers EO of *Vitex pseudo-negundo* Iranian plant [4]. However, some reports demonstrated that sabinene (20.3%) and *β*-caryophyllene (14.1%) were the major components of the flower EO of *Vitex pseudo-negundo* [28].

The major chemical compositions of the fruit EO of *Vitex pseudo-negundo* were *α*-pinene (32.7%), *α*-terpinyl acetate (16.8%), limonene (9.5%), and (E)-caryophyllene (8.3%) which accounted for almost 67%. The findings of this study partially agreed with the investigated EO composition of *Vitex pseudo-negundo* fruit harvested in Iran which reported *α*-pinene (25.4–47.7%), *α*-terpinyl acetate (0.5–21.1%), (E)-caryophyllene (4.6–24.7%), and limonene (8.9–13.9%) as major constituents of the fruit EO [4]. In research on *Vitex pseudo-negundo* collected from the Aydın/Koçarlı roadside in Turkey, the main constituents of EOs were different in comparison with the results of the present study. It was found that 1,8-cineole (8.24%), propenamide (6.07%), caryophyllene (5.56%), bicyclogermacrene (5.51%), sabinene (5.37%) were the main EO compounds [29]. Changes in the EOs chemical composition are common which is influenced by factors including, environmental conditions, geographical origin, genetic factors, and the plant growth stage. In this study, the EO compositions obtained from different plant organs were the same, especially in the main constituents, but there were considerable differences in the number of main compounds. More specifically, the EO composition of the *Vitex pseudo-negundo* leaf was quite similar to fruit EO composition containing *α*-pinene> *α*-terpinyl acetate > limonene> (E)-caryophyllene. The order of main compounds quantification in the *Vitex pseudo-negundo* flower was *α*-pinene> *α*-terpinyl acetate> (E)-caryophyllene > limonene.

### 3.2. Antifungal Activities of the EOs

The antifungal activities of the *Vitex pseudo-negundo* EOs against the tested fungi are shown in Table 2.

Based on the results, the leaf EO inhibited the growth of all the studied standard yeasts in the concentration range of 2–64 *μ*l/mL. Also, the flower and fruit EOs also inhibited the growth of the tested yeasts in the concentration range of 4–32 *μ*l/mL. The leaf EO had a fungicidal activity in the range of 8–64 *μ*l/mL on the tested yeasts, except for *C. albicans* and *C. parapsilosis*. In addition, all three EOs of leaf, flower, and fruit inhibited the growth of *Cryptococcus neoformans* at the concentration of 2, 4, and 4 *μ*l/mL, respectively.

The minimum inhibitory concentrations of leaf, flower, and fruit of *Vitex pseudo-negundo* EOs on the three clinically sensitive strains of *C. albicans* were 32–64 *μ*l/mL, 16–64 *μ*l/mL, and 8–32 *μ*l/mL. Also, the MIC of the leaf, flower, and fruit EOs on the three clinically resistant *C. albicans* strains were 32–64 *μ*l/mL, 16–64 *μ*l/mL, and 32 *μ*l/mL, respectively (Table 2). No significant difference in MIC was observed between resistant and sensitive strains of *C. albicans*. *Candida* species are common causes of nosocomial infections which can produce a wide range of infections. In this study, the EOs of the *Vitex pseudo-negundo* organs had inhibitory effects on *Candida* yeast growth in the range of 2–64 *μ*l/mL. The EOs of the *Vitex pseudo-negundo* organs inhibited the growth of clinical fluconazole-resistant strains of *C. albicans. Cryptococcus neoformans* is one of the most important pathogenic fungi, particularly in AIDS patients. In this study, the EOs at concentrations of 2–4 *μ*l/mL inhibited growth and at 8 *μ*l/mL concentration resulted in the elimination of this yeast. All of the *Aspergillus* standard strains were resistant to *Vitex pseudo-negundo* leaf and flower. The only fruit of the *Vitex pseudo-negundo* EO had a minimum inhibitory concentration on *Aspergillus* standard strains at a concentration of 64 *μ*l/mL. In the current study, the growth of the clinical dermatophytes isolates including *Microsporum gypseum, Epidermophyton floccosum,* and *Trichophyton rubrum* were inhibited by the *Vitex pseudo-negundo* leave and fruit EOs at a concentration range of 2–64 *μ*l/mL.

**Figure 1:**
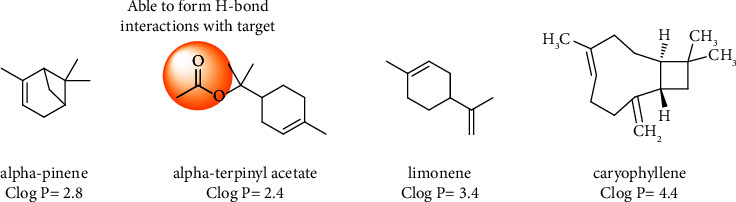
Chemical structures of some major compounds identified in the EOs of *Vitex pseudo-negundo* in Iran.

In this study, the EOs of *Vitex pseudo-negundo* exhibited antimicrobial activities against those examined fungal pathogens. The broad-spectrum antimicrobial property of the EOs might be attributable to their main compounds and their synergic effects. The chemical structures of major compounds of the *Vitex pseudo-negundo* EOs are shown in Figure 1.

*α*-Pinene (C_10_H_16_) is one of the most abundant terpene in nature known with a turpentine odor and colorless liquid. *α*-Pinene is used as a powerful antimicrobial agent due to subcellular size and good permeability into the cell (partition coefficient of octanol to water with log *P* = 2.8) which induces toxic effects on microorganism membranes and prevents antimicrobial efflux [30, 31]. It was also reported that *α*-pinene was capable of inhibiting phospholipase and esterase in yeasts [32]. These unique biochemical and biophysical properties of *α*-pinene and high content *α*-pinene EOs make them outstanding antimicrobial agents used in disinfectants [32, 33]. The high antimicrobial potency of EOs containing *α*-pinene was also seen in other studies. It was demonstrated that the MIC value of *α*-pinene was 360, 117, and 3125 *μ*l/mL against *Rhizopus oryzae, Cryptococcus neoformans,* and *C. albicans*, respectively. Interestingly, synergistic activities of *α*-pinene antimicrobial drugs result in around an 8-fold reduction in MIC value [32].

*In vitro* studies of pure *α*-terpinyl acetate have not been performed yet. *Chamaecyparis obtusa* EO containing high amounts of *α-terpinyl acetate* was used for the treatment of urinary tract infection. They concluded that this EO showed strong antifungal activity against *Pityrosporum ovale* (MIC = 5 *μ*l/mL) and *Saccharomyces cerevisiae* (MIC = 5 *μ*l/mL) [34]. *Elettaria cardamomum* EO showed great potency against Gram-positive bacteria and fungi as compared to Gram-negative bacteria. This may be due to the high contents of *α*-terpinyl acetate as oxygenate monoterpene and its synergic effects [35]. It was suggested that *α*-terpinyl acetate could participate in key hydrogen bond interactions with microorganisms and induce conformational cell changes. Besides, favorable log P (partition coefficient) value facilitates its partition into the lipid bilayer of the cell and induces its mode of action resulting in the death of the microorganism.

The microbial inactivation mechanism of limonene (4-isopropenyl-1-methylcyclohexene), as a natural hydracarbonic monoterpene, was due to sublethal damages on the outer membrane and an increase in the lethal effect. It should be noted that (+)-limonene can reach maximum cell permeability (∼90%) after 24 h incubation at pH 4.0 and 7.0 and induce inhibitory microorganism effects at very low concentrations [36]. A more recent study indicated the MIC values of 3 mg/ml for limonene against *Cryptococcus neoformans* [37]. Limonene is a major constituent of *Citrus maxima* (31.85% limonene) and *Citrus sinensis* (90.66% limonene). These EOs induced a promising fungitoxic spectrum against all tested fungi and caused 100% mycelial growth inhibition of the *A. fumigatus, A. terreus, A. alternata, Fusarium oxysporum,* and *Trichoderma viride* [38].

The *in vitro* antifungal evaluations confirmed that the fruit exhibited higher inhibitory activity, with geometric means-minimal inhibitory concentration (GM-MIC) values of 8 *μ*g/ml against tested yeast species compared with two other EOs by GM-MIC = 16 *μ*g/ml of flower EO and GM-MIC = 17 *μ*g/ml of leaf EO. However, the amount order of four major compounds in EOs is leaf (77.8%) > flower (72.3%) > fruit (67.3%). These data may indicate that the potency was related to not only the amounts of major compounds in the EOs but also the type and chemical structure of compounds [39]. Moreover, the importance of miner compounds and their synergic effects should not be neglected. Statistical analysis confirmed that the amount of oxygen-containing monoterpenes and sesquiterpenes was 29.21% which belonged to the fruit followed by 21.75% of the leaf and 21.67% of the flower. Considering these facts, the antifungal activities of EOs were more related to the effects of oxygenated natural compounds on the tested strains. Recent studies demonstrated that oxygenated monoterpenes possess strong antifungal activity *via* form H-bound interactions with the fungal membrane, deplete the ATP pool, and finally degrade microorganisms. Besides, the high solubility power of oxygenated monoterpenes in the media was another critical factor that justifies better antifungal activity of mentioned monoterpene [39]. To the best of our knowledge, there are a few studies to evaluate the antibacterial effect of *Vitex pseudo-negundo* and none of them evaluate the antifungal and antibiofilm activity of the mentioned EOs. In 2017, Balasubramani reported the considerable antibacterial activity of *Vitex pseudo-negundo* with MIC of 3.28, 21.26, and 21.07 *μ*l/mL against *Escherichia coli*, *Enterobacter aerogenes,* and *Enterococcus faecalis*, respectively [40].

### 3.3. Antibiofilm Activity of the EOs

Biofilm formation is a vital virulence factor in the *Candida* genus pathogenicity. Previous studies indicated that the presence of *Candida* in the form of biofilms might be related to the increased resistance to antifungal therapies. In addition, the formation of biofilm by *Candida* species has an important role in resistance to antifungal drugs, and the creation of this biofilm structure protects microbial cells against the host's immune defense.

In the present study, inhibitory impact of the tested EOs on *C. albicans* biofilm formation was determined by the XTT method. As shown in Table 3, the *Vitex pseudo-negundo* EOs inhibited the biofilm formation of *C. albicans*.

**Figure 2:**
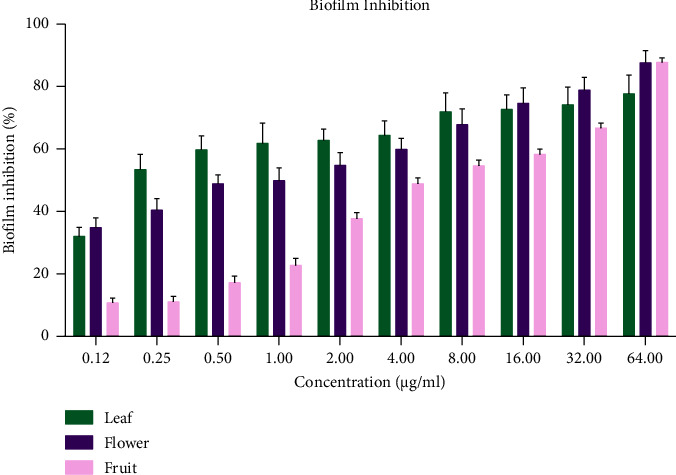
Biofilm inhibition of leaf, flower, and fruit of *Vitex pseudo-negundo* EOs.

Based on the results (Figure 2), EOs of leaf, flower, and fruit of this plant at concentrations of 0.25 *μ*l/mL, 1 *μ*l/mL, and 4 *μ*l/mL respectively, caused 50% inhibition of *C. albicans* biofilm formation. Moreover, the *Vitex pseudo-negundo* EOs almost completely inhibited the formation of biofilm by the *C. albicans* at a concentration of 64 *μ*l/mL. These actions may be related to the presence of a variety of constituents.

EOs are characteristically lipophilic and can penetrate the microbial cell membrane. Several studies have demonstrated that terpenes alter cell permeability by penetrating between the lipid bilayer membrane, producing holes and gaps in the membrane, disrupting lipid structure, and changing cell membrane flexibility [24, 41]. This phenomenon will in return result in major surface and morphological misshaping followed by reducing the adherence potency to the surface. Since the adherence process represents a major step in biofilm formation, therefore, reduced adherence will prevent biofilm formation. Also, it was reported that EOs components have an antibiofilm mechanism through the inhibition of glucan production and reduction of glucosyltransferase activity as well as downregulation of the glucans synthase genes [42]. In the present study, it was observed that the aerial part of *Vitex pseudo-negundo* in 64 *μ*l/mL concentrations effectively inhibited the biofilm formation of *C. albicans* in comparison with untreated control.

## 4. Conclusions

The data obtained in the present study showed that *Vitex pseudo-negundo* essential oils (EOs) have significant antimicrobial activity against the studied fungal species. The *Vitex pseudo-negundo* fruit EO was the most effective in inhibiting yeast growth. Additionally, the biofilm formation of *Candida albicans* was inhibited by the EOs extracted from the leaf, flower, and fruit. It is noteworthy that, considering the increasing issue of microbial resistance, these antimicrobial agents can also be used to control fungal infections and biofilm formation on both biological and nonbiological surfaces. To build upon the demonstrated inhibition of biofilm production achieved through our research, further comprehensive investigations involving a larger number of microorganisms are necessary to expand this study in future research activities.

## Figures and Tables

**Table 1:** Chemical composition of *Vitex pseudo-negundo* EOs (percentage).

No	Component	RI^a^	Leaf	Flower	Fruit
1	Tricyclene	920	—	0.01	0.2
2	*α*-Thujene	927	0.1	0.1	0.2
3	*α*-Pinene	936	39.3	30.3	32.7
4	Camphene	948	0.1	0.1	0.1
5	Thuja-2,4(10)-diene	954	0.01	—	0.2
6	Sabinene	973	3.7	3.6	2.5
7	*β*-Pinene	977	0.5	0.5	0.5
8	Myrcene	991	2.9	2.7	1.2
9	*α*-Phellandrene	1006	1.1	0.9	0.3
10	*α*-Terpinene	1017	0.2	0.1	0.2
11	*p*-Cymene	1024	0.1	0.3	0.6
12	Limonene	1029	13.8	11.6	9.5
13	*β*-Phellandrene	1030	0.8	0.8	0.5
14	1,8-Cineole	1031	0.3	0.2	0.3
15	(E)-*β*-Ocimene	1046	0.1	0.1	—
16	ϒ-Terpinene	1057	0.3	0.2	0.5
17	*cis*-Sabinene hydrate	1066	0.05	0.01	0.04
18	Terpinolene	1088	0.6	0.6	0.5
19	Linalool	1099	0.6	0.6	0.4
20	*n*-Amyl isovalerate	1108	0.1	0.01	0.1
21	*trans*-*p*-Mentha-2,8-dien-1-ol	1120	0.05	0.03	0.1
22	*α*-Campholenal	1125	—	—	0.5
23	*trans*-Pinocarveol	1138	—	—	1.1
24	*cis*-Verbenol	1140	0.05	0.04	—
25	Pinocarvone	1161	—	—	0.4
26	p-Mentha-1,5-dien-8-ol	1165	0.04	0.02	0.4
27	Menthol	1171	—	0.01	—
28	Terpinene-4-ol	1176	0.7	0.4	0.6
29	*α*-Terpineol	1190	0.7	0.5	1.0
30	Myrtenal	1195	—	—	0.4
31	Verbenone	1208	—	0.01	0.01
32	*trans*-Carveol	1218	—	—	0.2
33	Citronellol	1227	0.1	—	0.2
34	Cumin aldehyde	1238	—	—	0.06
35	Carvone	1242	—	—	0.1
36	Geraniol	1253	0.1	0.1	—
37	Geranial	1270	—	—	—
38	Bornyl acetate	1284	0.2	0.2	0.5
39	Thymol	1293	0.05	—	—
40	Carvacrol	1302	—	—	0.7
41	(2E,4E)-Decadienal	1315	—	—	—
42	Methyl geranate	1323	0.2	0.2	—
43	*δ*-Elemene	1336	0.3	0.8	0.4
44	*α*-Terpinyl acetate	1351	17.5	17.3	16.8
45	Citronellyl acetate	1353	—	—	—
46	*β*-Bourbonene	1383	—	0.04	—
47	*β*-Elemene	1391	—	0.1	0.03
48	*α*-Gurjunene	1408	0.2	0.5	0.4
49	(E)-Caryophyllene	1420	7.2	13.1	8.3
50	trans-*α*-Bergamotene	1434	0.2	0.4	0.4
51	(Z)-*β*-Farnesene	1442	0.4	0.6	0.6
52	*α*-Humulene	1452	0.8	1.4	—
53	(E)-*β*-Farnesene	1456	—	0.04	0.9
54	allo-Aromadendrene	1459	0.5	0.8	0.8
55	Germacrene D	1479	0.1	0.2	0.1
56	*β*-Selinene	1484	—	—	0.1
57	Bicyclogermacrene	1495	2.7	5.8	3.9
58	*β*-Bisabolene	1507	0.2	0.4	0.4
59	ϒ-Cadinene	1512	0.1	0.2	0.2
60	*δ*-Cadinene	1522	0.1	0.2	0.2
61	(E)-Nerolidol	1564	—	0.1	—
62	Germacrene B	1565	0.04	0.1	—
63	Spathulenol	1576	0.3	0.3	2.5
64	Caryophyllene oxide	1581	0.3	0.4	1.9
65	Viridiflorol	1589	0.05	0.1	0.1
66	Longiborneol	1600	0.2	0.3	0.6
67	epi-*α*-Cadinol	1639	0.2	0.5	1.6
68	*β*-Eudesmol	1648	0.5	0.5	0.6
69	*α*-Eudesmol	1652	—	0.2	—
70	*α*-Bisabolol	1682	0.1	0.2	0.1

*Note.* RI, linear retention indices on DB-5MS column, experimentally determined using homolog series of *n*-alkanes.

**Table 2:** Antifungal effects of the *Vitex pseudo-negundo* EOs on the yeast and mold strains based on broth microdilution method.

Fungi		ATCC/CBS	Leaf		Flower		Fruit		Fluconazole
			MIC90 (*μ*l/mL)	MFC (*μ*l/mL)	MIC90 (*μ*l/mL)	MFC (*μ*l/mL)	MIC90 (*μ*l/mL)	MFC (*μ*l/mL)	MIC (*μ*l/mL)
Standard strains	*C. albicans*	A (10261)	64	>64	16	64	32	64	4
	*C. glabrata*	A (90030)	16	32	16	>64	8	32	32
	*C. dubliniensis*	A (8501)	32	64	32	64	4	32	2
	*C. krusei*	A (6258)	16	64	8	64	4	16	32
	*C. tropicalis*	A (750)	16	64	16	>64	8	64	8
	*C. parapsilosis*	A (4344)	32	>64	32	>64	16	64	4
	*Cryptococcus neoformans*	A (9011)	2	8	4	8	4	8	2
	*A. flavus*	A (64025)	R	R	R	R	64	—	64
	*A. clavatus*	C (514.65)	R	R	R	R	64	—	64
	*A. fumigatus*	A (14110)	R	R	R	R	64	—	64
	*T. rubrum*	—	4	32	—	—	4	32	—
	*M. gypseum*	—	64	64	—	—	64	>64	—
	*E. floccosum*	—	2	8	—	—	2	16	—

Azole-sensitive clinical isolates	*C. albicans*	S (608)	64	64	64	>64	32	64	8
	*C. albicans*	S (615)	64	64	64	64	16	64	4
	*C. albicans*	S (538)	32	64	16	>64	8	64	16

Azole-resistant clinical isolates	*C. albicans*	R (625)	64	64	64	64	32	>64	>64
	*C. albicans*	R (634)	64	64	64	64	32	64	>64
	*C. albicans*	R (2303)	32	64	16	64	32	64	>64

*Note.* ATCC: American Type Culture Collection, CBS: Centraal Bureau voor Schimmelcultures, R: resistance, S: sensitive, MIC: minimum inhibitory concentration, and MFC: minimum fungicidal concentration.

**Table 3:** Inhibition of *C. albicans* biofilm in the presence of different concentrations of *Vitex pseudo-negundo* by the XTT reduction assay.

Concentration (*μ*l/mL)	Inhibition (%)		
	Leaf	Flower	Fruit
0.0	0.0	0.0	0.0
0.12	32.2	35.00	10.8
0.25	53.7	40.06	11.1
0.5	60.0	49.09	17.3
1	62.1	50.09	22.9
2	63.1	55.06	37.9
4	64.7	60.23	49.1
8	72.3	68.09	54.9
16	72.3	75.06	58.6
32	74.5	79.28	67.0
64	78.8	88.02	88.2

## Data Availability

The data used to support the findings of this study were supplied by Vice-Chancellor for Research of Shiraz University of Medical Sciences under license. Requests for data access should be made to Kamiar Zomorodian, kzomorodian@gmail.com. The data of this study were extracted from the MSc thesis of Maryam Izadpanah.
